# Relationship between Continuum of Hurst Exponents of Noise-like Time Series and the Cantor Set

**DOI:** 10.3390/e23111505

**Published:** 2021-11-13

**Authors:** Maria C. Mariani, William Kubin, Peter K. Asante, Joe A. Guthrie, Osei K. Tweneboah

**Affiliations:** 1 Department of Mathematical Sciences, University of Texas at El Paso, El Paso, TX 79902, USA; mcmariani@utep.edu (M.C.M.); jguthrie@utep.edu (J.A.G.); 2 Computational Science Program, University of Texas at El Paso, El Paso, TX 79902, USA; wkubin@miners.utep.edu (W.K.); pkasante@miners.utep.edu (P.K.A.); 3 Department of Data Science, Ramapo College of New Jersey, Mahwah, NJ 07430, USA

**Keywords:** Cantor set, fractals, homeomorphism, detrended fluctuation analysis, Hurst exponent

## Abstract

In this paper, we have modified the Detrended Fluctuation Analysis (DFA) using the ternary Cantor set. We propose a modification of the DFA algorithm, Cantor DFA (CDFA), which uses the Cantor set theory of base 3 as a scale for segment sizes in the DFA algorithm. An investigation of the phenomena generated from the proof using real-world time series based on the theory of the Cantor set is also conducted. This new approach helps reduce the overestimation problem of the Hurst exponent of DFA by comparing it with its inverse relationship with α of the Truncated Lévy Flight (TLF). CDFA is also able to correctly predict the memory behavior of time series.

## 1. Introduction

A ternary Cantor set is a set built by removing the middle part of a series when divided into three parts and repeating this process with the remaining shorter segments. It is the prototype of a fractal [1]. A fractal is a geometric object that has similar statistical properties to itself on all scales. If a fractal object is successively magnified, it looks similar or exactly like the original shape of the fractal. A similar pattern exhibited at increasingly smaller scales is often known in fractal mathematics as self-similarity [2,3]. In time series, self-similar phenomena describe the event in which the dependence in the time series decays more slowly than an exponential decay. Typically, it follows a power-like decay [4]. Scaling methods exist for quantifying the power-law exponent of the decay function such as Rescaled Range Analysis (R/S), Detrended Fluctuation Analysis (DFA) and the Truncated Lévy Flight (TLF).

The Rescaled Range Analysis (R/S) method by Hurst subdivides integrated time series into adjacent segment sizes and examines the range (R) of the integrated fluctuations. Then, a measure of dispersion, usually standard deviation (S), is determined as a function of segment size. A power law governs the approximate relationship between the Rescaled Range Analysis’ statistic (R/S) and the segment size [5].

The Detrended Fluctuation Analysis (DFA) by Peng et al. (1994) is a technique that quantifies the same power-law exponent of the R/S method. Addressing difficulties in determining correct power-law exponents of the R/S method in non-stationary time series resulted in the introduction of the DFA. Unlike the R/S method, the DFA uses a local detrending approach (usually linear regression) in the segments of the integrated series. For time series with higher-order trends, polynomial fit replaces the linear regression approach of the DFA [6]. This provides its power-law exponents’ protection against effects of nonstationarity and pollution of time series by external signals while eliminating spurious detection of long memory [7]. Empirical evidence has shown that the DFA performs well compared to other variance scaling methods including the R/S methods when estimating power-law exponents.

Usually, characterizing stochastic processes empirically requires the study of determining asymptotic probability density distributions (pdf) and temporal correlations. Brownian motion models the evolution of a particle’s position over time with the assumption that the movement of the particle follows a diffusive process with Gaussian distribution. This model did not describe accurately real-world time series because kurtosis of associated pdf is greater than that of the Gaussian distribution [8,9]. The Truncated Lévy Flight (TLF) model originated as a means to address the difficulties of the Brownian motion for working in long-range correlation scales. The scaling exponent (0<α≤2) of the TLF measures the memory behavior in time series that follows a diffusion process with Gaussian and non-Gaussian distributions [10].

In [11], a clear comparison was made between DNA and economics by the authors, showing the underlining similarities that allow researchers to model seemingly different phenomena using the same or slightly modified models. In the same manner, these variance scaling models have the added advantage of being used to model long memory effects in different fields where stochastic processes occur [2,7]. Thus, be it DNA sequencing, financial markets, geophysical time series etc., scaling methods have been used to detect long/short memory behaviors.

Scaling approaches serve as means of characterizing the dependence of observations separated in time series dominated by stochastic properties. Applications with DFA have been done in DNA sequences [6,12,13], neural oscillations [14], detection of speech pathology [15], heartbeat fluctuation in different sleep stages [16], describing cloud breaking [17], gearbox fault diagnosis [18], analysis of fetal cardiac data [19], streamflow in the Yellow River Basin in China [20], evaluation near infrared spectra of green and roasted coffee samples [21], just to mention a few.

Empirical evidence has shown that the DFA has the tendency of overestimating the scaling exponent [2,22]. We have not come accross any literature at the moment that describes a definite approach in the segment division step of the DFA algorithm. However, we observe that estimates of power-law exponents are influenced by the scale of choice [23,24]. Our goal in this work is to propose a definite non-overlapping segment division approach in the DFA algorithm (CDFA) that utilizes the theory of the ternary Cantor set. We show that using this approach we are able to rightly determine the correct scaling exponent to detect the memory behavior of the time series as well as reducing the over-fitting nature of the DFA. This approach has the advantage of generalizing the segment division step in the DFA algorithm. The Hurst exponents obtained from the CDFA method are then compared with the exponents of the DFA and the TLF on real time series.

In Section 2, we present proof of the relationship between the continuum of Hurst exponents of the DFA and Cantor set. We also present the scaling methods TLF, DFA and CDFA in this section. In Section 3, we present results and discussions from our investigation noting that for noise-like time series, anti-persistence, white noise and persistence behavior in time series imply 0≤H<0.5, H=0.5 and 0.5<H≤1 respectively. The overestimation of DFA’s Hurst exponent decreasing with the Cantor scales is also discussed in this section. Section 4 concludes the paper.

## 2. Methods

### 2.1. The Truncated Lévy Flight (TLF)

We provide a brief overview of the Truncated Lévy Flight (TLF) model in this subsection. The most general representation of the Lévy stable distribution is denoted by the characteristic function:

(1) $$\begin{array}{c} K\left(q,\alpha \right)=\exp\left\{i\mu q\;-\;\sigma^{\alpha }|q{|}^{\alpha }\left[1\;+\;i\beta .sign(q).\phi (q,\alpha )\right]\right\} \end{array}$$

where,

$$\phi \left(q,\alpha \right)=\left\{\begin{array}{c} (2/\pi )\ln(q),\;\;\;\;\;\alpha =1\\ -\tan(\pi \alpha /2),\;\;\;\alpha \neq 1. \end{array}\right.$$

The stability exponent α∈(0,2] defines the asymptotic decay of the pdf. σ∈(0,∞) measures dispersion. Skewness parameter β∈[−1,1] measures asymmetry of the distribution. μ∈(−∞,∞) is a scalar which determines the “location” or shift of the distribution. The sign x is the signum function of x∈R defined as sign(x)=x/|x|. The problem is that the variance of the distribution in (1) is finite but is not stable. This is because, large cut-off *l* results in slow convergence and a smaller cut-off *l* may result in abrupt tail [8]. In [25], the author generated a TLF to address the convergence problem by using a decreasing exponential cut-off function. Thus, the process in Equation (1) is truncated to obtain the TLF given by:

(2) $$T\left(q,\alpha \right)=\left\{\begin{array}{cc} c\;K(q,\alpha ), & |q|\;\leq \;l\\ 0, & |q|\;>\;l \end{array}\right.$$

for some normalizing constant *c*, stability exponent α∈(0,2] and cut-off length *l*. The characteristic function of the TLF in Equation (2) is given by

(3) $$\ln\left[T(q,\alpha )\right]=\frac{2\pi Al^{-\alpha }t\;\left[1\;-\;{\left({\left(ql/\sigma \right)}^{2}\;+\;1\right)}^{\alpha /2}\cos\left(\alpha \arctan\left(ql/\sigma \right)\right)\right]}{\alpha \Gamma (\alpha )\sin(\pi \alpha )}.$$

To determine the best scaling exponent (α) from characteristic equation in (3), we adjust the values of *A*, the cut-off parameter *l* and the scaling exponent α simultaneously to fit the characteristic function to the data.

### 2.2. Detrended Fluctuation Analysis (DFA)

Given the noise-like time series ψ, we find the integrated series

(4) $$Y=\sum_{k}\left(\psi_{k}\;-<\psi >\right).$$

to determine the Root Mean Squared Fluctuations (RMSF) from Equation (5) below

(5) $$F\left(s\right)={\left\{\frac{1}{N}\sum_{j}{\left[Y_{j}-Y_{j}^{s}\right]}^{2}\right\}}^{1/2}$$

A log–log plot of the RMSF against the series length *s* produces a directly proportional relation given by

$$F\left(s\right)\propto s^{H}$$

(6) logF(s)−Hlog(s)=K,

where H:= Hurst exponent of the DFA and $H_{min}\leq H\leq H_{max}$ [4].

### 2.3. Cantor Detrended Fluctuation Analysis (CDFA)

In this subsection, we prove that the subspace $\left[H_{min},H_{max}\right]$ of Hurst exponents is homeomorphic to [0, 1] of the Cantor set. We also present an illustration of the Cantor set and the algorithm for the CDFA.

**Theorem** **1.** *A map $f:\left[H_{min},H_{max}\right]\to \left[0,1\right]$ between the topological spaces of Hurst exponents of noise-like time series and the Cantor set is a homeomorphism if it has the following properties:*

- *f* is a bijection;
- *f* is continuous;
- the inverse function $f^{-1}$ is continuous.

If two topological spaces admit a homeomorphism between them, we say they are homeomorphic: they are essentially the same topological space.

**Proof.**  Let $H_{min}\leq H\leq H_{max}$ and 0≤y=f(H)≤1, then the map $f:\left[H_{min},H_{max}\right]\to \left[0,1\right]$ gives

(7) $$H_{min}-H_{min}\leq H-H_{min}\leq H_{max}-H_{min}$$

(8) $$0\;\leq \;\frac{H-H_{min}}{H_{max}-H_{min}}\;\leq \;1.$$

Thus,

(9) $$y=f\left(H\right)=\frac{H-H_{min}}{H_{max}-H_{min}}.$$

Now, we need to prove that the map *f* is homeomorphic to the Cantor set.

The map f(H) is said to be bijective if and only if f(a)=f(b) for all *a*, *b* implies that a=b. From

$$f\left(a\right)=\frac{a-H_{min}}{H_{max}-H_{min}}\;\;\;and\;\;\;f\left(b\right)=\frac{b-H_{min}}{H_{max}-H_{min}},$$

f(a)=f(b)

$$⟹a-H_{min}=b-H_{min}$$

⟹a=b.

Thus, the map f(H) is a bijection.

The map f(H) is continuous at some value *c* in its domain if f(c) is defined, the limit of *f* as *H* approaches *c* exists and the function value of *f* at *c* equals the limit of *f* as *H* approaches *c*. The function f(c) is defined as

(10) $$f\left(c\right)=\frac{c-H_{min}}{H_{max}-H_{min}}.$$

The limit of *f* as *H* approaches *c* equals

(11) $$\lim_{H\to c^{+}}f\left(H\right)=\lim_{H\to c^{-}}f\left(H\right)=\frac{c-H_{min}}{H_{max}-H_{min}}.$$

The left- and right-sided limits are equal from (11). Therefore,

(12) $$\lim_{H\to c}f\left(H\right)=\frac{c-H_{min}}{H_{max}-H_{min}}.$$

Hence we observe that the right hand side of Equation (10) is equal to right hand side of Equation (12). Thus, it follows that

$$\lim_{H\to c}f\left(H\right)=f\left(c\right)=\frac{c-H_{min}}{H_{max}-H_{min}}.$$

Thus, the map *f* is continuous at some value H=c for a differentiable fractal.

The inverse function of *f* (i.e.,$f^{-1}\left(H\right)$) exists.

(13) $$y=f\left(H\right)=\frac{H-H_{min}}{H_{max}-H_{min}}$$

(14) $$\left(H_{max}-H_{min}\right)y=H-H_{min}$$

(15) $$H=H_{min}+\left(H_{max}-H_{min}\right)y$$

Interchanging *H* and *y* gives

(16) $$y=f^{-1}\left(H\right)=H_{min}+\left(H_{max}-H_{min}\right)H,$$

the inverse function of f(H).

The inverse map $f^{-1}$ is continuous at some value *s* in its domain if $f^{-1}\left(s\right)$ is defined, the limit of $f^{-1}$ as *H* approaches *s* exists and the function value of $f^{-1}$ at *s* equals the limit of $f^{-1}$ as *H* approaches *s*. $f^{-1}\left(s\right)$ is defined as

(17) $$f^{-1}\left(s\right)=\left(1-s\right)H_{min}+sH_{max}.$$

The limit of $f^{-1}$ as *H* approaches *s* equals

(18) $$\lim_{H\to s^{+}}f^{-1}\left(H\right)=\lim_{H\to s^{-}}f^{-1}\left(H\right)=H_{min}+\left(H_{max}-H_{min}\right)s.$$

(19) $$\Rightarrow \;\;\;\lim_{H\to s}f^{-1}\left(H\right)=H_{min}+\left(H_{max}-H_{min}\right)s.$$

Since the right hand side of Equation (17) equals the right hand side of Equation (19) it implies that,

$$\lim_{H\to s}f^{-1}\left(H\right)=f^{-1}\left(s\right)=\left(1-s\right)H_{min}+sH_{max}.$$

Thus, the inverse map $f^{-1}$ exists and is continuous at some value H=s.

Therefore, the map f(H) is a homeomorphism and $H\in [H_{min},H_{max}]$ is homeomorphic to [0,1] of the Cantor set for noise-like time series. □

#### 2.3.1. Illustration of the Cantor Set

**Figure 1:**
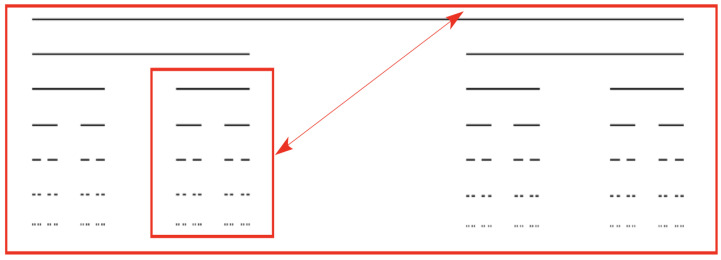
Fractal behavior of a ternary Cantor set.

In this subsection, we take real-world noise-like time series and remove middle thirds up to four (4) levels so that it is similar to the Cantor set. This phenomenon is depicted in Figure 1 [26]. It shows that the segments appear the same at different scales in successive magnifications of the Cantor set from levels $C_{0}$ to $C_{6}$. $C_{0}$ depicts the original time series with no missing parts and $C_{6}$ represents the remaining time series after removing middle thirds for the sixth time. For the sake of experimentation, we limit our scope to levels from $C_{0}$ to $C_{3}$.

#### 2.3.2. Definition

The subset of intervals of the Cantor set is defined recursively as:

1. $C_{0}=\left[0,1\right]$;
2. $C_{1}=\left(\frac{1}{3},\frac{2}{3}\right)$;
3. $C_{n}=\frac{C_{n-1}}{3}\cup \left(\frac{2}{3}+\frac{C_{n-1}}{3}\right)$ for n≥2.

The ternary Cantor set is defined as $C=\left[0,1\right]\backslash \left(\cup_{n=1}^{\infty }C_{n}\right)$. The level $C_{0}$ indicates the interval we begin with. For $C_{1}$, [0,1] is divided into 3 sub-intervals and the middle sub-interval $\left(\frac{1}{3},\frac{2}{3}\right)$ is removed. For $C_{2}$, each of the remaining intervals from $C_{1}$ are divided into 3 sub-intervals and their middle sub-intervals $\left(\frac{1}{9},\frac{2}{9}\right)$ and $\left(\frac{7}{9},\frac{8}{9}\right)$ are removed. This procedure can continue indefinitely by removing open middle third sub-interval of each interval obtained in the previous level. Due to issues with the dimension of the Cantor sets (i.e., dimension of 0.631 < 1), we rescale the integrated series $\psi_{t}$ by dividing each observation by the maximum data point:

$$\psi_{t}=\frac{\Psi_{t}}{\max(\Psi_{t})}.$$

s.t. $\psi_{t}\in \left[0,1\right]$.

#### 2.3.3. Algorithm of the CDFA

Here, we present a modification of the DFA algorithm called CDFA to generalize the segment division step of the DFA. The CDFA algorithm consists of four (4) main steps:

1. given the time series $\psi_{t}$ of length *N*, find the integrated series shifted by the mean <ψ>,
  $$Y_{j}=\sum_{i=1}^{j}\left(\psi_{i}\;-<\psi >\right).$$
2. the cumulatively summed series $Y_{j}$ is then segmented into equal non-overlapping segments of various sizes Δs. Δs is based on the Cantor set theory scale ($\Delta s=3^{n}$, n≥0). The number of non-overlapping segments is calculated as:
  $$N_{\Delta s}\equiv int\left(\frac{N}{\Delta s}\right)=int\left(\frac{N}{3^{n}}\right).$$
  The Cantor set scaling function is computed for multiple segments to highlight both slow- and fast-evolving fluctuations that control the structure of the time series.
3. Root Mean Squared Fluctuation (RMSF) is computed for multiple scales of the integrated series:
  $$F\left(\Delta s\right)\equiv {\left\{\frac{1}{2N_{\Delta s}}\sum_{j=1}^{2N_{\Delta s}}{\left[Y_{j}-Y_{j}^{\Delta s}\right]}^{2}\right\}}^{1/2}$$
  where *j* denotes the sample size of segments $N_{\Delta s}$. We compute RMSF from j=1 to $2N_{\Delta s}$ not $N_{\Delta s}$. We sum from beginning to end and from end to beginning, then an average of the values is calculated so that every data point is considered. Conversely, the large segments interweave many local periods with both small and large fluctuations and therefore average out their differences in magnitude.
4. the least squares regression fit of F(Δs) versus the Cantor scales Δs on a log–log scale produces the power-law notation computed for multiple scales:
  $$F\left(\Delta s\right)\propto {\left(\Delta s\right)}^{H^{c}}$$
  $$log\left(F\left(\Delta s\right)\right)=H^{c}log\left(\Delta s\right)+log\left(C\right),$$
  where $H^{c}:=$ Hurst exponent of the CDFA which measures memory behavior in the noise-like time series.

#### 2.3.4. Real Time Series

**Figure 2:**
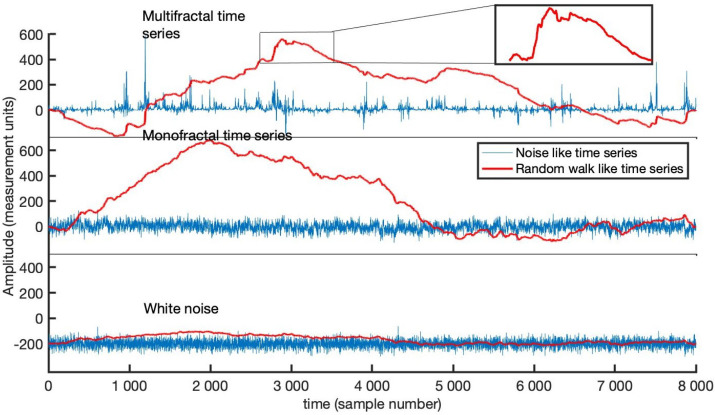
Biomedical time series plots.

In Figure 2, the time series multifractal (upper panel), monofractal (middle panel) and white noise (lower panel) used in the experiment are noise-like biomedical time series with 8000 rescaled sample data points each [27]. The red trajectory depicts the random walk of the respective series. Observe that the fractal depicted by the multifractal time series at the peak looks very similar to the entire monofractal time series. Thus, comparing the series in the upper panel to the middle panel, the multifractal series has many fractals compared to the one for the monofractal series. We determine DFA’s Hurst exponents for the remaining series after removing the middle thirds of each series at each level. It should be noted that the white noise time series has a structure independent of time with Hurst exponent close to H=0.5 whereas noise-like monofractal and multifractal time series exhibit persistence behavior s.t. 0.5<H≤1.

## 3. Results

Below are tables of results of power-law exponents from the implementation of the DFA algorithm on the respective time series. Note from Figure 1 that

1. $C_{0}$ denotes the entire time series (with 8000 data points) and produces one Hurst exponent $H_{1}$ shown in row 1 of Table 1, Table 2 and Table 3;
2. $C_{1}$: after removing middle third of the time series for the first time, we have two (2) set of data with respective data points 2666 and 2667 and corresponding Hurst exponents $H_{1}$ and $H_{2}$ as shown in row 2 of Table 1, Table 2 and Table 3;
3. $C_{2}$: we have four (4) data sets remaining after deleting middle third for the second time. The data sets have 888, 889, 888 and 889 observations, respectively. Thus, we obtain four (4) Hurst exponents, namely, $H_{1},\;H_{2},\;H_{3}$ and $H_{4}$ as shown in row 3 of Table 1, Table 2 and Table 3;
4. $C_{3}$: deleting middle third for the third time produces eight (8) partitions of data with 296 data points each. Each of the data sets produces a Hurst exponent resulting in eight (8) exponents $H_{1},\;H_{2},\;H_{3},\;H_{4},\;H_{5},\;H_{6},\;H_{7},$ and $H_{8}$ in total. These exponents are shown in the last rows of Table 1, Table 2 and Table 3.

It should be noted that the same data points from partitioning correspond to the white noise, monofractal, and multifractal time series. The Hurst exponents also follow respectively.

From Table 1, we observe closeness of the Hurst exponents of the white noise series to H=0.5 for all levels from $C_{0}$ to $C_{3}$. This confirms the phenomena that are exhibited in the fractal nature of the Cantor set in white noise time series. No matter how many sections of a white noise series are removed, the left-over series still exhibits similar characteristics as the whole white noise series.

Table 2 present Hurst exponents between 0.5 and 1 (0.5<H≤1) for long memory monofractal time series for levels $C_{0},C_{1},C_{2}$ and $C_{3}$. The phenomena exhibited in the monofractal time series from the table above are similar to the fractal nature of the Cantor set. The series left behind after removing the middle thirds of the monofractal time series exhibits similar statistical properties as the whole.

Hurst exponents of the multifractal time series lie within the range 0.5<H≤1 for all levels $C_{0},C_{1},C_{2}$ and $C_{3}$ from Table 3. This illustrates the fractal phenomena depicted by the Cantor set where successive magnification of the Cantor produces a copy of itself. This can be seen in Figure 1. Thus, self-similar behavior persists after removing the middle thirds of the whole series up to the level $C_{3}$. Results from Table 1, Table 2 and Table 3 confirm that successive magnification of noise-like time series shows a similar pattern at increasingly smaller scales. Thus, the statistical characteristics of part of noise-like series are similar to that of the whole. This phenomenon is commonly known in fractals as self-similarity.

**Figure 3:**
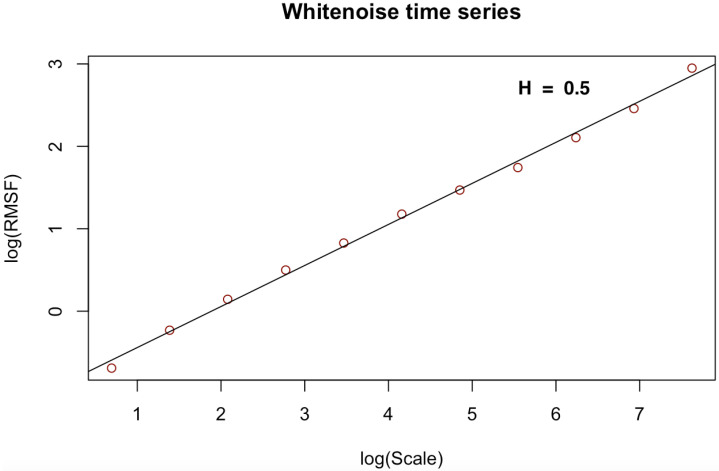
Log–log fit of white noise time series using DFA.

**Figure 4:**
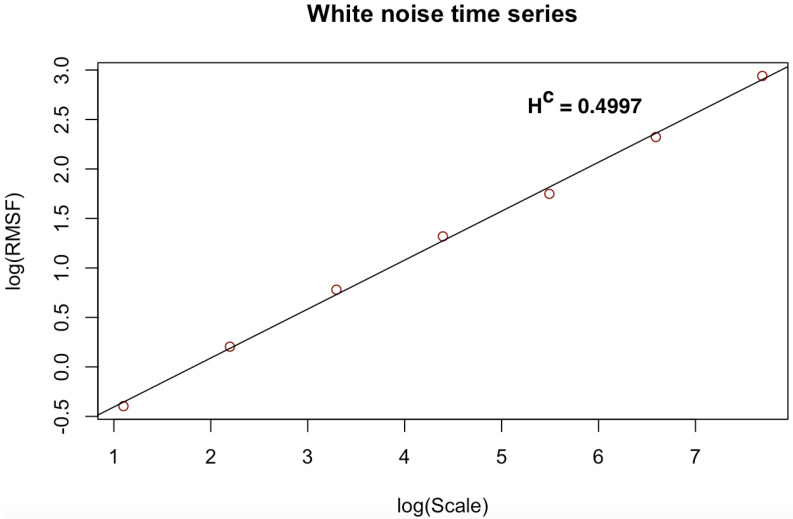
Log–log fit of white noise time series using CDFA.

**Figure 5:**
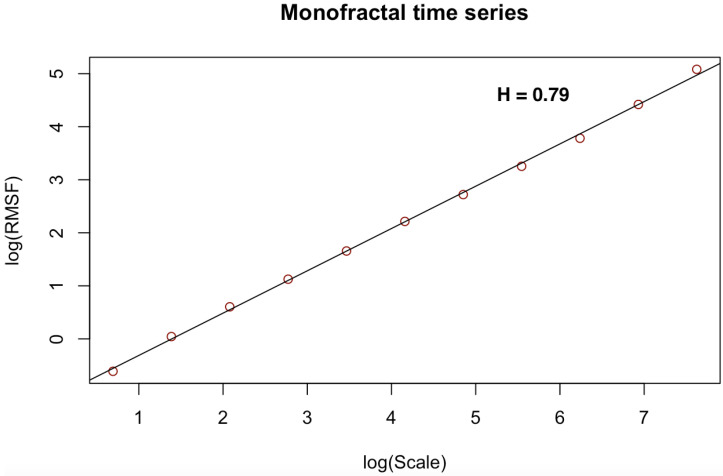
Log–log fit of monofractal time series using DFA.

**Figure 6:**
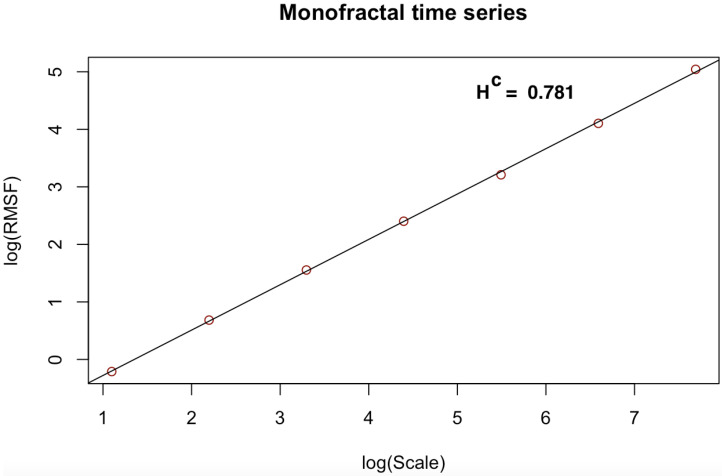
Log–log fit of monofractal time series using CDFA.

**Figure 7:**
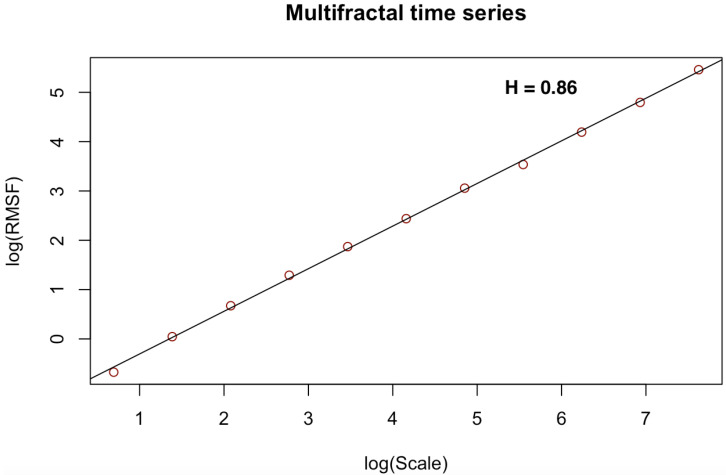
Log–log fit of multifractal time series using DFA.

**Figure 8:**
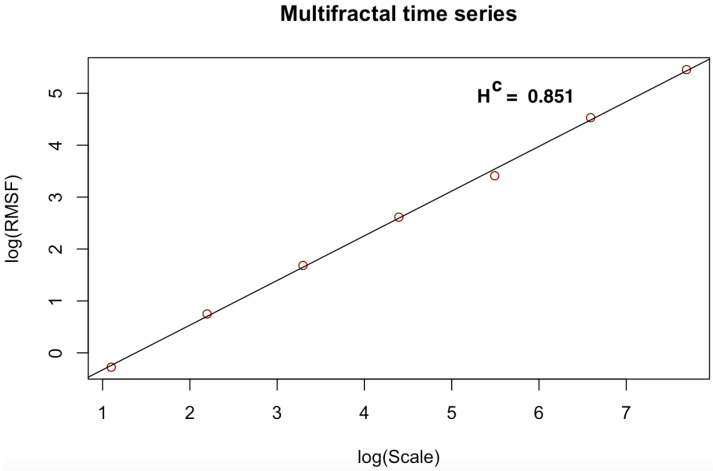
Log–log fit of multifractal time series using CDFA.

Figure 3, Figure 4, Figure 5, Figure 6, Figure 7 and Figure 8 shows the log–log fits of RMSF and scales of the white noise, monofracal and multifractal bio-medical series using the DFA and the CDFA. The first two (2) plots (i.e., Figure 3 and Figure 4) present fits of the white noise using the DFA and CDFA. The next two (2) plots (i.e., Figure 5 and Figure 6) illustrate the fit of monofractal series using the DFA and CDFA. The last two(2) plots (i.e., Figure 7 and Figure 8) show fits of the multifractal series using the DFA and the CDFA.

Table 4 above has six (6) columns of results in total. The first column (H) represents the Hurst exponents of the DFA, the second column $\left(H^{c}\right)$ denotes the Hurst exponents of the CDFA and the difference between the exponents in the first two columns are found in the third column. The column for α denotes the scaling exponents of the TLF. The last two columns represent the multiplication of the Hurst exponents of the DFA (H) and the scaling exponent of the TLF (α), as well as the multiplication of the Hurst exponents of the CDFA $\left(H^{c}\right)$ and the scaling exponents of the TLF. Upon investigating Hurst exponents of white noise, monofractal and multifractal time series using the DFA and CDFA, we observe differences in their exponents, as shown in Table 4. Hurst exponent of white noise time series changes slightly but that of the monofractal and multifractal time series changes about 1%. The slight changes in the exponents are a result of subdividing the time series as multiples of 3 (ternary base) at each level using the CDFA. This helps to curb the problem of overestimation associated with DFA. Notwithstanding the differences between the exponents, they still depict the same processes modeled herein (i.e., noise-like time series). The exponent of the white noise is close to 0.5 whereas that of the noise-like monofractal and multifractal series lie within the range 0.5<H≤1, depicting long-memory behavior.

## 4. Discussion

The results obtained from the Table 1, Table 2, Table 3 and Table 4 suggest that segment size may not always be hard-coded in the DFA algorithm based on the length of the time series in question. Especially for time series with odd lengths, the process can be automated using the fractal phenomena of the Cantor set to obtain equal segment sizes and satisfactory Hurst exponents.

Furthermore, multiplying Hurst exponents of the DFA and CDFA with the scaling exponents (α) of the Truncated Lévy flight (TLF) suggests that $H^{c}$ is a better estimate. For the monofractal and multifractal noise-like time series, we observe that $H^{c}\alpha$ is approximately equal to 1 while Hα exceeds 1. This deviates from the inverse relationship between the Hurst exponents and the scaling exponents of the TLF for Gaussian noise as discussed in the paper [4]. This highlights the overestimation of the Hurst exponent of the DFA approach that happens in practice.

## 5. Conclusions

In this work, we have proposed a modification to the DFA algorithm by utilizing the theory of the tenary Cantor set in the segment division step. The Cantor DFA (CDFA) has been compared to the α exponent of the truncated Lévy model and the Hurst exponent of the DFA. We have in addition proved that the interval of the Hurst exponent of the DFA is homeomorphic to the Cantor set. We confirm the results from the proof by illustrating the fractal phenomena exhibited by the Cantor set using real-world time series in Table 1, Table 2 and Table 3.

Our results from numerical simulations show that the CDFA generates better estimates of Hurst exponents. The CDFA proposed in this work automates the segment sizes in the DFA algorithm using the number base 3 theory of the Cantor set, where the time series is divided into multiples of 3 at each level. This modification helps to curb the overestimation problem of the Hurst exponent (*H*) of the DFA by determining segment sizes based on the fractal phenomena depicted by the Cantor set while correctly predicting the memory behavior of the series in question.

The results are shown in Table 4 where the Hurst exponent of the CDFA is compared with that of the DFA and the scaling exponents (α) of the TLF. In [4], a relationship was established between the Hurst exponent of the DFA and the α exponent of the TLF. The CDFA is also shown to satisfy this relation, thus making it possible to extract the α exponent of the TLF from the Hurst exponent of the CDFA.

The CDFA approach can be applied to time series with odd lengths, time series whose lengths are not easily divisible by even numbers, time series whose lengths do not permit equal segmentation, etc. These kinds of series exist in several industries, including financial, geophysics, health and the like. Another application of the CDFA would be to act as a control model for the ordinary DFA to reduce the chances of overestimation of the Hurst exponent.

Since this is a modification of the DFA, there is the need to simulate CDFA with different data sets having varying characteristics for which the DFA has been shown to correctly detect their scaling behavior. An example will be DNA sequences, financial markets, etc., for further comparison of the model performance against the DFA.

For future work, we seek to investigate the robustness of the CDFA as stated earlier by simulating the model with data sets from different fields, including, but not limited to, DNA sequences, financial markets and geophysical data. In the case of “big data”, we seek to extend the CDFA by “parallelizing” the sequential code of the CDFA (PCDFA) to improve its efficiency in simulation.

## Figures and Tables

**Table 1:** DFA’s Hurst Exponents of White noise time series.

Levels	Hurst Exponents
$C_{0}$	$H_{1}=0.50$
$C_{1}$	$H_{1}=0.50,\;H_{2}=0.45$
$C_{2}$	$H_{1}=0.54,\;H_{2}=0.45,\;H_{3}=0.52,\;H_{4}=0.42$
$C_{3}$	$H_{1}=0.50,\;H_{2}=0.54,\;H_{3}=0.4,\;H_{4}=0.49,\;H_{5}=0.59,\;H_{6}=0.43,\;H_{7}=0.42$, $H_{8}=0.57$

**Table 2:** DFA’s Hurst exponents of monofractal time series.

Levels	Hurst Exponents
$C_{0}$	$H_{1}=0.79$
$C_{1}$	$H_{1}=0.80,\;H_{2}=0.68$
$C_{2}$	$H_{1}=0.81,\;H_{2}=0.69,\;H_{3}=0.74,\;H_{4}=0.68$
$C_{3}$	$H_{1}=0.65,\;H_{2}=0.80,\;H_{3}=0.63,\;H_{4}=0.72,\;H_{5}=0.78,\;H_{6}=0.68,$ $H_{7}=0.67,\;H_{8}=0.79$

**Table 3:** DFA’s Hurst exponents of multifractal time series.

Levels	Hurst Exponents
$C_{0}$	$H_{1}=0.86$
$C_{1}$	$H_{1}=0.86,\;H_{2}=0.75$
$C_{2}$	$H_{1}=0.75,\;H_{2}=0.88,\;H_{3}=0.70,\;H_{4}=0.78$
$C_{3}$	$H_{1}=0.82,\;H_{2}=0.69,\;H_{3}=0.77,\;H_{4}=0.97,\;H_{5}=0.69,\;H_{6}=0.70,$ $H_{7}=0.91,\;H_{8}=0.90$

**Table 4:** Comparison of scaling exponents of DFA (H) & CDFA $\left(H^{c}\right)$ & TLF (α) on noise-like time series.

Time Series	***H***	$H^{c}$	Difference	α	***H*** α	$H^{c}\alpha$
White noise	0.5	0.4997	0.0003	1.97	0.985	0.9844
Monofractal	0.79	0.781	0.009	1.28	1.0112	0.9997
Multifractal	0.86	0.851	0.009	1.17	1.0062	0.9976

## Data Availability

The data used in this work can be accessed at https://www.ntnu.edu/inb/geri/software.

## Abbreviations

The following abbreviations are used in this manuscript:

DFA	Detrended Fluctuation Analysis
CDFA	Cantor Detrended Fluctuation Analysis
TLF	Truncated Lévy Flight
R/S	Rescaled Range Analysis
